# Facilitating informed choice about non-invasive prenatal testing (NIPT): a systematic review and qualitative meta-synthesis of women’s experiences

**DOI:** 10.1186/s12884-018-2168-4

**Published:** 2019-01-14

**Authors:** Alexandra Cernat, Chante De Freitas, Umair Majid, Forum Trivedi, Caroline Higgins, Meredith Vanstone

**Affiliations:** 1 Honours Life Sciences BSc Program, McMaster University, Hamilton, ON Canada; 2 Health Sciences Education Program, McMaster University, Hamilton, ON Canada; 3 Department of Health Research Methods, Evidence & Impact, McMaster University, Hamilton, ON Canada; 4 Health Quality Ontario, Toronto, ON Canada; 5 Department of Family Medicine, McMaster University, DBHSC 5003E, 100 Main St W, Hamilton, ON L8P 1H6 Canada; 6 Centre for Health Economic and Policy Analysis, McMaster University, Hamilton, ON Canada

**Keywords:** Non-invasive prenatal testing, Prenatal screening, Qualitative meta-synthesis, Informed decision-making

## Abstract

**Background:**

Non-invasive prenatal testing (NIPT) can be used to accurately detect fetal chromosomal anomalies early in pregnancy by assessing cell-free fetal DNA present in maternal blood. The rapid diffusion of NIPT, as well as the ease and simplicity of the test raises concerns around informed decision-making and the potential for routinization. Introducing NIPT in a way that facilitates informed and autonomous decisions is imperative to the ethical application of this technology. We approach this imperative by systematically reviewing and synthesizing primary qualitative research on women’s experiences with and preferences for informed decision-making around NIPT.

**Methods:**

We searched multiple bibliographic databases including Ovid MEDLINE, EBSCO Cumulative Index to Nursing & Allied Health Literature (CINAHL), and ISI Web of Science Social Sciences Citation Index (SSCI). Our review was guided by integrative qualitative meta-synthesis, and we used a staged coding process similar to that of grounded theory to conduct our analysis.

**Results:**

Thirty empirical primary qualitative research studies were eligible for inclusion. Women preferred to learn about NIPT from their clinicians, but they expressed dissatisfaction with the quality and quantity of information provided during counselling and often sought information from a variety of other sources. Women generally had a good understanding of test characteristics, and the factors of accuracy, physical risk, and test timing were the critical information elements that they used to make informed decisions around NIPT. Women often described NIPT as easy or just another blood test, highlighting threats to informed decision-making such as routinization or a pressure to test.

**Conclusions:**

Women’s unique circumstances modulate the information that they value and require most in the context of making an informed decision. Widened availability of trustworthy information about NIPT as well as careful attention to the facilitation of counselling may help facilitate informed decision-making.

**Trial registration:**

PROSPERO 2018 CRD42018086261.

**Electronic supplementary material:**

The online version of this article (10.1186/s12884-018-2168-4) contains supplementary material, which is available to authorized users.

## Background

Non-invasive prenatal testing (NIPT) for chromosomal anomaly represents a significant evolution of prenatal screening technology. Commercially available to many since 2011, NIPT assesses cell-free fetal DNA present in maternal blood to screen pregnancies for common chromosomal anomalies, either through a quantitative “counting” method that uses targeted parallel sequencing, or by identifying maternal and fetal allele distributions using single-nucleotide polymorphisms (SNPs) [1]. It is most often used to detect aneuploidy in chromosomes 13, 18, 21, and the sex chromosomes. The SNP-based NIPT can also identify five clinically significant microdeletions [2], and clinical applications are expanding rapidly [3].

Testing with NIPT can be done as early as 9 to 10 weeks of pregnancy, up until the time of birth. It is more accurate than other forms of prenatal screening, with a sensitivity ranging from 90 to 99% and specificity ranging from 99 to 100% depending on the condition [4]. When compared to other prenatal screening tests, it carries a low false positive rate of 0.09 to 0.13% depending on the condition [5]. NIPT is not accurate enough to be considered a diagnostic test [6]. Since it is non-invasive, it is not associated with iatrogenic pregnancy loss [7].

NIPT has been commercially available in Hong Kong and the United States since 2011, and has quickly spread to over 60 countries [8]. The price of the test varies internationally and depends on the anomalies being tested for. In Canada, NIPT costs between CAN $550–$795 [9], while in the United States, prices range from US $600–$800 [10]. In the United Kingdom, the test costs from £375–£1500 [11]; elsewhere in Europe the price is typically between €260–€770 [12–14].

Increasing numbers of health insurance companies and publicly funded health systems are providing coverage for NIPT, with most reimbursing women who are at high risk for chromosomal anomalies. “High risk” is variously defined, and often operationalized using maternal age, history, or positive results from other screening tests [15]. In North America, some Canadian provinces adopted this model of coverage as early as 2014, with others not providing any coverage. In the United States, the majority of insurance companies fund NIPT for high-risk pregnancies, though several have expanded their coverage to all pregnant women [10]. In some jurisdictions such as Hong Kong and Singapore, NIPT is available only through a private-pay system [16, 17]. In Europe, Denmark, France, the Netherlands, and Switzerland offer public funding for NIPT, contingent with risk [18]. In the United Kingdom, reimbursement is only provided in the context of a 2-year study that began in 2018 [18], but the National Screening Committee has recommended that screening with NIPT be done for high-risk pregnancies [19]. To date, Belgium is the only country to publicly fund NIPT as a first-tier or primary test [18].

NIPT has attracted significant attention, in part due to its rapid diffusion, but also because of the potential to disrupt traditional prenatal testing pathways and its myriad ethical implications. NIPT is not a diagnostic test, and as a result most clinical practice guidelines recommend that all positive NIPT results should be confirmed with invasive fetal diagnostic testing, as well as that no irrevocable decisions about a pregnancy should be made on the basis of NIPT alone [6, 7, 20]. However, the introduction of NIPT has been associated with a decreased uptake of diagnostic testing [21], and some clinicians have reported a decrease in the number of invasive diagnostic procedures performed since NIPT was introduced [22].

Research on women’s preferences about NIPT shows high levels of support for this technology [23–32] as well as a strong desire that it be available to all women due to its accuracy and safety [28, 30, 33, 34]. Despite these benefits, NIPT raises several ethical issues. For instance, because NIPT is able to provide accurate information about fetal sex early in a pregnancy, one concern is that test results could be used for sex selective termination [35, 36]. In countries without universal public funding for NIPT, there may be inequity of access to the test and subsequent prenatal care opportunities [36–38]. The accuracy and early timing of NIPT results may also have implications for the disabled community, in that the number of people born with a disability may decrease over time, leading to increased discrimination and stigmatization, as well as diminished availability of resources and supports [39].

The simplicity and ease of the test also raises concerns about informed decision making and the potential for routinization. Informed decisions are those that are founded upon relevant knowledge and are concordant with a person’s values [40]. They enable a person to exercise autonomy, which is regarded in the ethical literature as intrinsically valuable [40]. Promoting reproductive autonomy is recognized as one of the core principles of prenatal testing [41]. While NIPT can facilitate a woman’s reproductive autonomy by providing her with accurate information risk-free and early in her pregnancy, these same elements of the test could also lead to an erosion of informed choice and a reduced ability to exercise autonomy [40, 41]. Because NIPT is a simple procedure that poses no risk to the fetus, there is the danger that women may view it as just “another blood test” [42, 43] and therefore opt-in without fully understanding its importance or implications [41]. The rapid diffusion and widespread implementation of NIPT also raises concerns that health care providers may not yet be fully prepared to counsel women appropriately due to a lack of time and a lack of confidence in counselling about this new technology [34, 41]. An additional challenge to informed decision making exacerbated by NIPT is that it could lead to greater societal pressure for women to undergo prenatal testing [40, 42, 43]. Because of the accuracy of results, and the lack of physical risk creating the perception that there is no disadvantage to testing, women may feel that they are expected to have NIPT [43]. For these reasons, it is important to understand how to facilitate informed, autonomous decisions about NIPT. This includes understanding women’s values as they consider their prenatal care, how they make decisions around their pregnancy, and what their preferences are for education, resources, and support during this time.

This review answers the following research questions: *How do women experience informed decision making about NIPT? How do they use information and negotiate between different aspects of the test to make a decision? What are their preferences for the facilitation of informed choice?* There is a burgeoning amount of empirical literature [44–48] about the challenges of informed decision making about NIPT. The purpose of this review is to bring together this collection of primary research to comment on women’s perspectives, experiences, and preferences for informed decision making about NIPT.

## Methods

We performed a systematic review of primary qualitative research about NIPT as part of a Health Quality Ontario (HQO) health technology assessment (HTA) on NIPT. Detailed methods are available in that report (Vanstone M, Cernat A, Majid U, De Freitas C, Trivedi F. Women’s and clinician perspectives on non-invasive prenatal testing (NIPT): a systematic review and qualitative meta-synthesis, forthcoming). The systematic review and meta-synthesis conducted for HQO described the experiences of women, clinicians, and others with rich lived experience of the test (e.g. parents of children with conditions detected by NIPT). For the current paper, we focus on the literature about women’s and their partners’ experiences, preferences, and values pertaining to making informed decisions about NIPT.

### Literature search

The search strategy (Appendix 1) was developed in partnership with medical librarians [see Additional file 1 for Appendix 1]. We combined a topic-specific search with a validated filter designed to identify qualitative research [49]. We searched Ovid MEDLINE, EBSCO Cumulative Index to Nursing & Allied Health Literature (CINAHL), and ISI Web of Science Social Sciences Citation Index (SSCI) for studies published from January 1, 2007 to September 21, 2017. We updated the search monthly until August 1, 2018 and incorporated eligible studies into the analysis as they were identified.

Included studies were English-language papers reporting primary qualitative empirical research, including the qualitative component of mixed-methods studies, that involved adult women who had personal experience with NIPT. We only included research published in peer-reviewed journals (i.e. no theses) and given the emerging nature of the technology, we did not limit the search based on the location of the study. We excluded studies not in English, animal and in vitro studies, studies that did not include primary data, studies that were quantitative or labelled “qualitative” but did not use a qualitative descriptive or interpretive methodology (e.g. quantitative content analysis, structured surveys), as well as editorials, clinical case reports, or commentaries. Studies addressing topics other than NIPT, and those that did not focus on the perspectives of women with experience of NIPT (e.g. studies of public opinion) were also excluded. To ensure consistency between reviewers during the literature sorting process, AC, CDF, UM, FT, and MV first met to discuss the inclusion and exclusion criteria, establish procedural guidelines, and sort a practice library. AC, CDF, UM, FT, and MV participated in the sorting process, with at least two reviewers reviewing each title and abstract to ensure concordance between decisions about the eligibility of each article. When discrepancies were identified between the two reviewers, a third reviewer was invited to adjudicate. If the eligibility was still unclear, the entire research team discussed the article in question to come to a consensus opinion. Full text articles were obtained when the title and abstract alone were not sufficient to establish eligibility.

Our review was guided by the technique of integrative qualitative meta-synthesis [50–52], also known as qualitative research integration, which begins with an a priori research question answered by a systematic review and an integrative analysis of findings from each eligible study.

AC, CDF, UM, FT, and MV participated in data extraction and analysis. Our data consisted of the findings from both the results and discussion sections of the relevant studies. Qualitative findings are the authors’ interpretations of their own data – the “data-driven and integrated discoveries, judgments, and/or pronouncements researchers offer about the phenomena, events, or cases under investigation” [52]. Primary data makes ad hoc appearances in qualitative studies; we did not focus our analysis on participant quotes but we did extract excerpts when useful for illustrative purposes. Data was extracted by one author and then verified by a second author.

We used a staged coding process similar to that of grounded theory [53, 54], in which findings from multiple articles are broken into their conceptual components, summarized and subsequently re-grouped according to their thematic relationships. First, the included studies were divided into four groups. AC, CDF, UM and FT, and MV each performed initial line-by-line coding on one set of papers and identified preliminary categories. The analytic group met to discuss which categories would be used for the next round of focused coding. These categories were formed based on the prevalence of information across multiple studies, and the relevance of that information to our research questions. Broader themes emerged from these preliminary categories; in subsequent rounds of coding AC, CDF, UM, FT, and MV each coded all 30 of the included studies while focussing on a particular theme or themes. We used a constant and iterative approach, comparing categories with research findings, raw data excerpts, and our interpretations of the studies. Analysts reviewed each other’s coding work as part of preparation for regular meetings to discuss the iterative process of analysis, compare findings and interpretations, and decide the next analytic steps. This technique allowed us to organize and reflect on the full range of descriptive and interpretive insights across the entire body of research [52, 55]. The resulting analysis reflects the range of findings while retaining the original meaning of each study, offering a new integrative interpretation which both describes findings across the studies and interprets meaning from the collective body of literature.

Within the field of qualitative research, there is a lack of consensus on the importance of, and methods or standards for, critical appraisal of research [56, 57]. In other publications, we have detailed our philosophical objections to critical appraisal of qualitative research for the purpose of evidence syntheses (Vanstone M, Cernat A, Majid U, De Freitas C, Trivedi F. Women’s and clinician perspectives on non-invasive prenatal testing (NIPT): a systematic review and qualitative meta-synthesis, forthcoming) [56]. Briefly, critical appraisal tools rely on structured quality criteria, potentially to do the disservice of studies created within different philosophical paradigms, or for different purposes. High quality qualitative research is not guaranteed if high quality procedures are followed, and with external constraints such as journal word counts, there is no guarantee that high quality procedures will be reported comprehensively, even if followed. The usefulness, originality, and contribution of each paper relies on aspects of the research which may be difficult to judge from what is reported in a manuscript, and on factors which are difficult to report, such as the analytic ability and prowess of the researcher. For this review, we presumed that the academic peer review and publication processes eliminated scientifically unsound studies, according to current standards.

## Results

**Fig. 1:**
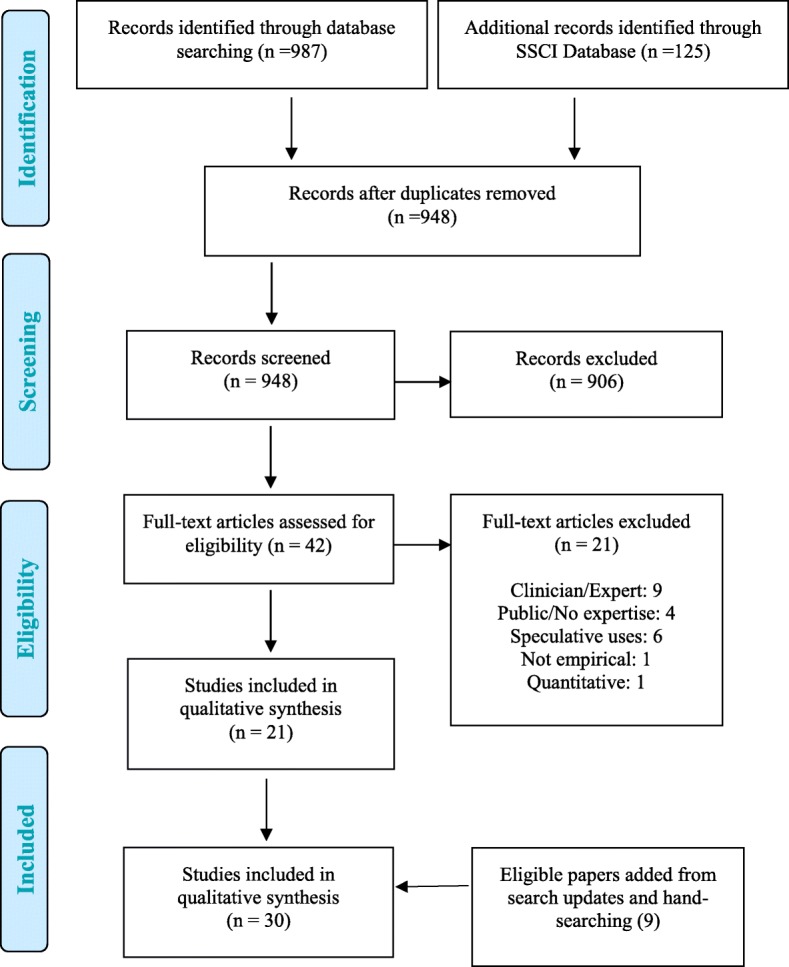
Citation flow chart

Our systematic search yielded 948 studies (with duplicates removed). We also conducted a hand-search of reference lists of all included articles and continued to retrieve relevant hits from monthly search updates. In total, 30 studies were eligible for inclusion. These included studies involved 1237 patients and family members with experience with NIPT. Figure 1 illustrates the search process, while Tables 1, 2, 3 and 4 describe the included studies, including the study design, geographic location, and number and type of participants.

**Table 1:** Summary of Included Qualitative Studies

Authors	Date	Title	Country	Qualitative Methodology	Participants	Main Research Question or Purpose of Study
Agatisa PK, Mercer MB, Leek AC, et al. [23]	2015	A first look at women’s perspectives on noninvasive prenatal testing to detect sex chromosome aneuploidies and microdeletion syndrome	United States	Interpretive Description	31 women	What are women’s knowledge and attitudes about the use of NIPT to detect sex chromosome aneuploidies? What are their views on emerging applications of NIPT to detect microdeletion syndromes?
Agatisa PK, Mercer MB, Mitchum A, et al. [65]	2018	Patient-centered obstetric care in the age of cell-free fetal DNA prenatal screening	United States	Grounded Theory and Adapted Approaches	27 women	What are patients’ perspectives on the clinical infrastructures required to their educational and decision-making needs around prenatal genetics and genomics?
Chen A, Tenunen H, Torkki P, et al. [72]	2017	Considering medical risk information and communicating values: a mixed-method study of women’s choice in prenatal testing	Finland	Thematic Analysis and Adapted Approaches	26 women	How do women decide between NIPT, CVS, and amniocentesis when all three are publicly covered for high-risk pregnancies?
Crombag NMTH, van Schendel R, Schielen PCJI, et al. [69]	2016	Present to future: what the reasons for declining first-trimester combined testing tell us about accepting or declining cell-free DNA testing	Netherlands	Content Analysis	46 women	Why do women decline first-trimester combined screening? How do these reasons relate to future (hypothetical uptake of cell-free DNA testing?
Daley R, Hill M, & Lewis C. [75]	2017	Evaluation of patient information leaflets or non-invasive prenatal testing for Down’s syndrome	United Kingdom	Thematic Analysis and Adapted Approaches	81 women	To develop and validate a patient information leaflet to support the introduction of NIPT into the prenatal screening program in the UK.
Farrell RM, Mercer MB, Agatisa PM, et al. [24]	2014	It’s more than a blood test: patients’ perspectives on noninvasive prenatal testing	United States	Grounded Theory and Adapted Approaches	53 women	How do women understand the utility of NIPT compared to other prenatal screening modalities? What factors do they consider in their decision-making process?
Farrell R, Hawkins A, Barragan D, et al. [64]	2015	Knowledge, understanding, and uptake of noninvasive prenatal testing among Latina women	United States	Not Specified	25 women	What factors influence the knowledge, understanding, and uptake of NIPT among Latina women? How do they use information from NIPT to make decisions about their pregnancy?
Farrell RM, Agatisa PK, Mercer MB, et al. [66]	2015	Balancing risks: the core of women’s decisions about noninvasive prenatal testing	United States	Grounded Theory and Adapted Approaches	53 women	How do women understand the benefits and risks of NIPT? How does this understanding influence their views of the benefits and risks of prenatal screening and testing?
Floyd E, Allyse, MA, & Michie M. [25]	2016	Spanish- and English-speaking pregnant women’s views on cfDNA and other prenatal screening: practical and ethical reflections	United States	Grounded Theory and Adapated Approaches	24 women	How are the views of Latina and non-Latina women about cfDNA screening and other prenatal screening similar and different?
Gross NEZ, Geva-Eldar T, Pollak Y, et al. [58]	2017	Attitudes toward prenatal genetic testing and therapeutic termination of pregnancy among parents of offspring Pradr-Willi syndrome	Israel	Not Specified	85 parents (male and female)	What are the attitudes of parents of offspring with Prader-Willi syndrome towards prenatal diagnostic testing and t termination of pregnancy in hypothetical pregnancies where Prader-Willi syndrome is expected?
Haidar H, Vanstone M, Laberge AM, et al. [26]	2018	Cross-cultural perspectives on decision-making regarding non-invasive prenatal testing: A comparative study of Lebanon and Quebec	Canada and Lebanon	Qualitative Description	17 women and 16 partners	How do women in Quebec and Lebanon make decisions around NIPT?
How B, Smidt A, Wilson NJ, et al. [61]	2018	‘We would have missed out so much had we terminated’: what fathers of a child with Down syndrome think about current non-invasive prenatal testing for Down syndrome	Australia	Thematic Analysis and Adapted Approaches	5 fathers	What are the views of Australian fathers towards the availability of NIPT in relation to their lived experience of parenting their child with Down syndrome?
Kibel M & Vanstone M. [33]	2017	Reconciling ethical and economic conceptions of value in health policy using the capabilities approach: a qualitative investigation of non-invasive prenatal testing	Canada	Content Analysis	38 women	Using NIPT as a case study, can the capabilities approach be used to resolve contradictions between economic and ethical framings of ‘value’ for morally challenging health technologies?
Lau JYC, Yi H, & Ahmed S. [63]	2016	Decision-making for non-invasive prenatal testing for Down syndrome: Hong Kong Chinese women’s preferences for individual vs. relational autonomy	China	Thematic Analysis and Adapted Approaches	36 women	What are Hong Kong Chinese women’s preferences around individual vs. relational autonomy in decision-making about NIPT for Down syndrome?
Lewis C, Hill M, Skirton H, et al. [29]	2012	Fetal sex determination using cell-free fetal DNA: service users’ experiences of and preferences for service delivery	United Kingdom	Thematic Analysis and Adapted Approaches	38 women and 6 partners	What are users’ experiences of NIPT? What decisions did they make upon receiving NIPT results? What are their preferences for how the test should be offered in clinical practice?
Lewis C, Hill M, Skirton H, et al. [59]	2012	Non-invasive prenatal diagnosis for fetal sex determination: benefits and disadvantages from the service users’ perspective	United Kingdom	Grounded Theory and Adapted Approaches, and Thematic Analysis	38 women and 7 partners	What are users’ perspectives on the benefits and disadvantages of NIPT?
Lewis C, Silock C, & Chitty LS. [30]	2013	Non-invasive prenatal testing for Down’s syndrome: pregnant women’s views and likely uptake	United Kingdom	Thematic Analysis and Adapted Approaches	40 women	What are pregnant women’s views and preferences for NIPT?
Lewis C, Hill M, & Chitty LS. [27]	2016	A qualitative study looking at informed choice in the context of on-invasive prenatal testing for aneuploidy	United Kingdom	Thematic Analysis and Adapted Approaches	45 women	How do women experience informed consent for NIPT?
Lewis C, Hill M, & Chitty LS. [28]	2016	Women’s experiences and preferences for service delivery of non-invasive prenatal testing for aneuploidy in a public health setting: a mixed methods study	United Kingdom	Thematic Analysis and Adapted Approaches	81 women	What are women’s experiences with and preferences for NIPT?
Li G, Chandrasekharan S, & Allyse M. [68]	2017	“The top priority is a healthy baby”: narratives of health, disability, and abortion in online forum discussions in the US and China	United States and China	Content Analysis	Not specified; used online forums	How are the views of Chinese and American individuals around prenatal screening similar and different?
Long S, O’Leary P, Lobo R, et al. [71]	2018	Women’s understanding and attitudes towards Down’s syndrome and other genetic conditions in the context of prenatal screening	Australia	Thematic Analysis and Adapted Approaches	30 women	How do women understand current prenatal screening modalities? What do women understand about Down syndrome and other genetic conditions that can be detected through prenatal screening?
Mozersky J. [74]	2015	Hoping someday never comes: deferring ethical thinking about noninvasive prenatal testing	United States	Grounded Theory and Adapted Approaches	Not specified	To illustrate that pre-test counselling for NIPT may not include an adequate discussion of ethical concerns.
Piechan JL, Hines KA, Koller DL, et al. [67]	2016	NIPT and informed consent: an assessment of patient understanding of a negative NIPT result	United States	Interpretive Content	98 women	How do patients understand NIPT and interpret negative results? How do they perceive NIPT in comparison to other prenatal screening modalities? How do they understand the advantages and limiations of NIPT?
Reese KM, Czerwinski J, Darilek S, et al. [31]	2018	Attitudes toward and uptake of prenatal genetic screening and testing in twin pregnancies	United States	Thematic Analysis and Adapted Approaches	42 women	What factors play a role in women’s decision around prenatal genetic screening in twin pregnancies?
van Bruggen MJ, Henneman L, & Timmermans DRM. [73]	2018	Women’s decision making regarding prenatal screening for fetal aneuploidy: a qualitative comparison between 2003 and 2016	Netherlands	Thematic Analysis and Adapted Approaches	41 women	What are the similarities and differences in pregnant women’s decision-making process around prenatal screening in 2003 compared with 2016?
van Schendel RV, Kleinveld JH, Dondorp WJ, et al. [32]	2014	Attitudes of pregnant women and male partners towards non-invasive prenatal testing and widening the scope of prenatal screening	Netherlands	Content Analysis	41 women and 19 male partners	What are the attitudes of pregnant women and their male partners toward NIPT? What are their views on expanding the scope of prenatal screening through NIPT?
van Schendel RV, Kater-Kuipers A, van Vliet-Lachozki EH, et al. [60]	2017	What do parents of children with Down syndrome think about non-invasive prenatal testing (NIPT)?	Netherlands	Content Analysis	21 women and 6 family members	What are the attitudes of parents of children with Down syndrome towards NIPT?
Vanstone M, Yacoub K, Giacomini M, et al. [70]	2015	Women’s experiences of publicly funded non-invasive prenatal testing in Ontario, Canada: considerations for health technology policy-making	Canada	Grounded Theory and Adapted Approaches	38 women	What are women’s experiences with publicly funded NIPT?
Vanstone M, Cernat A, Nisker J, et al. [34]	2018	Women’s perspectives on the ethical implications of non-invasive prenatal testing: a qualitative analysis to inform health policy decisions	Canada	Grounded Theory and Adapted Approaches	38 women	How do women understand the ethics implications of the implementation of NIPT in Ontario, Canada?
Yi H, Hallowell N, Griffiths S, et al. [62]	2013	Motivations for undertaking DNA sequencing-based non-invasive prenatal testing for fetal aneuploidy: a qualitative study with early adopter patients in Hong Kong	China	Thematic Analysis and Adapted Approaches	45 women	What are women’s motivations for undertaking NIPT screening for Down syndrome? What are their perceptions about the testing process?

**Table 2:** Body of Evidence Examined According to Study Design

Study Design	Number of Eligible Studies
Thematic Analysis and Adapted Approaches	12
Grounded Theory and Adapted Approaches	7
Content Analysis	5
Not Specified	2
Interpretive Content Analysis	1
Interpretive Description	1
Qualitative Description	1
Multiple	1
Total	30

**Table 3:** Body of Evidence Examined According to Study Location

Study Location	Number of Eligible Studies
United States of America	9
United Kingdom	6
Netherlands	4
Canada	3
China	2
Multiple Locations	2
Australia	2
Finland	1
Israel	1
Total	30

**Table 4:** Body of Evidence Examined According Type and Number of Participants

Type of Participants	Number of Participants
Patient	1093
Partners or Family Members	144
Total^a^	1237

^a^Two studies [68, 74] did not identify the number of participants

Through our staged coding process, we identified a wide variety of themes related to informed decision making, such as pre-test counselling and availability of information, test accuracy, procedural ease, safety of NIPT, and test timing and return of results. These themes are interconnected and we synthesize them by discussing two main elements of informed choice: first, women’s access to information on NIPT, and second, women’s understanding and use of that information. We then outline potential threats to informed choice such as routinization and a pressure to test, and present some of women’s preferences that may help safeguard and facilitate informed choice.

Of those studies that specified their number and type of participants, six included both patients and their partners or family members [26, 29, 32, 58–60]. We report on *women’s* access, information, and preferences because these studies amalgamated their findings into *participant* views, rather than distinguishing the source – patient or family member – of each perspective or experience [26, 29, 32, 60], or they focused predominantly on women’s perspectives [59]. These studies did not report tension between women and their partners or relatives regarding NIPT. One paper included only male partners [61]; their views were consistent with those of women in other studies, but where applicable we have indicated that the given views are shared by both women and their partners.

### Women’s access to information

In order for a decision to be considered informed, a woman requires comprehensive and understandable information about NIPT. By *access*, we refer to how women obtained such information and how they learned about NIPT. In other words, access refers to the informational sources women relied on or sought out, and how reliable and trustworthy they perceived those sources to be.

Across the studies included in this review, women went to their clinicians, academic institutions [62], the media [27, 62], online sources such as discussion groups and blogs [62, 63], and friends or family members who had had experience with NIPT [63, 64] to learn about the test. They largely preferred that their information come from clinician counselling [23, 24, 30, 63, 65, 66], but many discussed needing to research the technology themselves to obtain what they considered to be an adequate knowledge base [23, 30, 63]. Women in one UK study, all of whom received in-person counselling with a dedicated NIPT research midwife, expressed a clear preference to receive counselling from a midwife, as they felt midwives have a strong understanding of the different prenatal testing options, are knowledgeable about the different conditions detected, and are generally seen for prenatal care [28]. Others in the same study felt that the first clinician they saw during pregnancy was the most appropriate person to provide counselling [28].

Despite a common preference for learning about NIPT from their clinician, women in many studies were dissatisfied with their counselling experience because they felt that their clinician was not sufficiently informed about the technology to facilitate informed choice [23, 25, 29, 33, 34, 60, 62, 63]. Women felt that the gap in health care provider knowledge was especially pronounced regarding the experience of raising a child with conditions such as trisomies 13 and 18, and sex-linked disorders, which are all detected by NIPT [59, 60, 63]. Some parents of children with Down syndrome attributed this lack of information in counselling to health care providers’ unfamiliarity with the condition [60].

In addition to a perceived lack of clinician knowledge, appointment time constraints also contributed to women’s dissatisfaction with counselling. Women strongly agreed that consultations were too short for adequate counselling about NIPT to be possible, and noted that as a result of short appointments they often had a variety of questions and concerns that went unaddressed [63]. However, in some cases women felt as though *too much* information was received at once [24, 65, 67]. This was described as an information overload that could make women feel overwhelmed and therefore hinder their ability to contextualize and prioritize information for decision making [24, 65].

It is important to note that women’s experiences and satisfaction with counselling seemed to be modulated by the type of clinician they saw. Women who felt they received adequate counselling to make an informed choice were more likely to have seen or to prefer seeing specialized health care providers such as genetic counsellors and specialized nurses [29, 34] and specialist physicians [30]. In contrast, family physicians or general practitioners [29, 34] and obstetricians [62] were more likely to be described as less knowledgeable. Women discussing NIPT with midwives had mixed impressions about the counselling they received. Women in two UK studies [28, 30] who had been introduced to NIPT by a midwife were very happy with how they were counselled and felt that midwives are very knowledgeable. However, women who had already developed trusting relationships with specialized physicians and genetic counsellors felt that, by comparison, midwives were not sufficiently well informed to advise about genetic testing options [29].

### Women’s understanding and use of information

Women conceptualized and prioritized different aspects of NIPT differently, and the way they valued and used information to make decisions about prenatal screening changed depending on their unique personal and social situations [26, 27, 68–70]. In general, women appreciated the advantages, disadvantages, limitations, and consequences of prenatal testing [24, 27, 63, 66, 71]. However, some misperceptions around the accuracy of NIPT compared with that of invasive diagnostic tests [25, 34, 70], as well as misperceptions around the difference between a screen and a diagnostic test, [25, 71] persisted. Across the studies in this review there was consistent evidence that the factors of accuracy, physical risk, and test timing were the critical information elements that women focused on and negotiated between to make informed decisions around NIPT and prenatal testing.

#### Accuracy

Many women understood that NIPT has high specificity and sensitivity [24, 25, 27, 30, 31, 60, 63, 66, 68, 70, 72], although there was some disagreement among them about the comparative accuracies of NIPT and traditional prenatal testing modalities [25–27, 30, 70]. A minority of women believed NIPT to be the most accurate prenatal testing option, but most identified invasive testing as being most definitive [25, 27, 30, 70].

Test accuracy played a crucial role in enabling women to discern between prenatal testing options [27, 29, 30, 32, 33, 63, 66, 70, 72]. Some women reported that the accuracy of NIPT was sufficiently high that they did not feel the need to confirm results with invasive diagnostic testing [25, 26, 32, 33, 63], and the high test accuracy and safety provided women and their partners with a sense of increased control over the pregnancy [23, 25–31, 33, 59–61, 65, 69, 72]. Other women considered that NIPT was accurate enough to merit using the technology, but felt that it does need to be confirmed by a diagnostic test that offers more definitive results [25, 26, 29, 70]. They considered the potential for uncertainty as a disadvantage because they could not use test results to make confident decisions about their pregnancy [24, 66]. Finally, a subset of women expressed a willingness to wait for invasive testing to become available before doing other prenatal screening because of the certainty of those results [70, 72]. However, this was very much a minority view.

#### Physical risk

For many women, the most important aspect of NIPT was the fact that it poses no physical risk to the fetus [24–31, 33, 59, 62, 63, 66, 70, 72, 73], with some women identifying this as their main decision-making factor between tests [62, 70, 72, 73]. Across the papers included in this review, the majority of women understood that an invasive diagnostic test such as amniocentesis or chorionic villus sampling was required to confirm the results of NIPT [25, 26, 29, 66, 70]. Despite this understanding, most women seemed to make decisions around prenatal screening and confirmatory testing by relying on their values, such as their views around termination, and balancing physical risk against test accuracy. For some women, especially those who did not intend to terminate, the risk-free nature of NIPT was paramount and they considered it sufficiently accurate to decline confirmation via diagnostic testing [26, 27, 66, 74]. These women considered NIPT to be an excellent alternative to amniocentesis rather than a precursor to it [24, 26, 30, 32, 33, 66, 70, 73]. Whether NIPT was understood as a substitute or precursor to invasive testing, it provided women with the opportunity to decline diagnostic testing which may have a risk of miscarriage. Some women stated the opportunity to avoid “uncomfortable, scary, and stressful” [70] diagnostic testing was the biggest benefit of NIPT. In this way, women who would have declined invasive testing because of the physical risk involved were still able to gain reliable information about their pregnancy which they could use to make more informed choices about the remainder of their prenatal care [23, 28, 29, 32, 33, 69, 72–74].

#### Test timing

Women who accessed NIPT in the first trimester of pregnancy were able to more thoroughly consider and establish priorities between accuracy, timing of test results, and personal risk of fetal chromosomal anomalies when compared to women who accessed the test later in pregnancy [70]. Obtaining test results early provided women with more time to make informed and thoughtful decisions about the best course of action for their prenatal health, and made them and their partners feel a greater degree of control over and satisfaction with their decisions [24, 29, 59, 61, 62]. For those women considering termination, early access to information was very important because it made the process much easier both physically and emotionally (i.e. the bond to the fetus was not as strong) [29, 30, 34, 59, 60, 70]. In contrast, women who accessed testing later in pregnancy valued fast return of results more than they did test accuracy and safety because they felt that their decisions about prenatal health were time-sensitive, and as a result were more inclined to opt for invasive testing [28]. The majority of women across the papers included in this review were very supportive of a universal offer of NIPT in the first trimester of pregnancy [28, 30, 33, 34], emphasizing that obtaining information earlier enabled them and their partners to better consider the pregnancy management and to prepare emotionally, physically, and financially for raising their child [24–29, 31, 33, 59–61, 65, 69, 72].

### Threats to informed choice

In general, women were very enthusiastic about NIPT [23, 25–32, 58], but their discussions around the ease of the testing procedure gave many authors [27, 30, 32, 62, 74] cause for concern that the simplicity of the technology could lead to routinization, pressure to test, and an erosion of informed choice.

NIPT is a simple procedure that many women described as convenient and “just another blood test” to be taken during pregnancy [24, 25, 27–30, 59, 68], with some expressing a preference for undergoing testing on the same day it was introduced in counselling [28]. This raises the concern that women may agree to NIPT without a sufficiently thorough consideration of possible outcomes and the potential for invasive testing or termination [24, 27, 28, 30, 32, 60]. Many authors used women’s descriptions of NIPT as simple and easy to explicitly identify the potential for routinization [27, 30, 32, 62, 74]. Health care providers and patients alike could view NIPT as “an ethically uncontentious procedure” [62] since it is “a new technology [...] masked behind a[n] old [one]” [27], and it could thus be integrated into prenatal care as a standard, routine test. To facilitate informed decision making, several authors recommended leaving time for reflection between the initial introduction of NIPT and the procedure itself [27, 30].

A second worry was that women could experience pressure to undergo NIPT and may therefore choose testing even if they wish to decline. This pressure stems from public perception of NIPT as an easy and risk-free test [24, 30, 32, 60] which makes women feel as though participation is expected [24]. Women also experience pressure to undergo NIPT from family members and partners [29, 30]. Increased stigma attached to conditions detected by NIPT [28, 32, 60, 73] and consenting to another form of screening earlier in the pregnancy [28] are also sources of pressure that may make women feel uncomfortable declining NIPT.

### Women’s preferences to facilitate better informed decision making

Women expressed that both the quality and type of information available about NIPT need to be improved and expanded to better facilitate informed decision making. As previously indicated, women made significant efforts to educate themselves using sources other than their clinicians. However, many were dissatisfied with these sources, questioning their trustworthiness [62] and expressing preference that health care providers [23–25, 28, 30, 63, 66], websites [23, 30], and support groups [75] should provide more information. In particular, women felt that there was insufficient and inadequate information available on the accuracy of different prenatal testing modalities [23, 25, 75], the sensitivity and specificity of NIPT [66], the implications of false positive or negative results [24, 25, 66, 75], and the potential next steps in the care pathway following NIPT results [24, 25]. However, the information women required extended beyond explanations of prenatal testing: women preferred that counselling also build a scientific foundation upon which they could then begin to assimilate, understand, and interpret information about NIPT. More specifically, they expressed desire for clearer discussions around risk ratios, probabilities, and detection rates of testing [62], as well as for more explicit comparisons of the accuracy of available prenatal testing modalities [63]. Women also wanted a better understanding of how to accurately interpret inconclusive results [28, 62], and identified the need for more information on parenting a child with the conditions NIPT is able to detect [24, 25]. This information was desired both pre- and post-test [66] in straightforward language without technical terms [64, 75]. Ideally it would be delivered through multiple mediums of communication, including: verbally either in person or over the phone [23, 24], in the form of a written leaflet or pamphlet [23, 30, 75], or online [23, 25, 30].

## Discussion

In order for a woman to make an informed decision around NIPT, she first requires access to high quality, accurate information about the technology. Across the studies included in our review, the majority of women preferred that this information come from their clinicians. However, most were disappointed by the counselling they received, particularly from family physicians, general practitioners, and obstetricians. In order to fill gaps in the information they desired, women sought information from a variety of other sources including the media, discussion groups, forums, and websites. Although many women were satisfied with their understanding of NIPT, several misconceptions still persisted, raising questions as to whether they were adequately informed about the test.

Facilitating informed decisions about NIPT is challenged by issues beyond obtaining high quality information. Given that NIPT is procedurally simple, consisting of a single blood test which may be experientially similar to the other blood tests required in pregnancy, there is a risk that informed choice may be challenged by routinization [76]. Routinization has been raised as a challenge to informed decisions about prenatal tests for decades; it refers to the potential for a prenatal screening test to become a normalized part of the prenatal care pathway, and correspondingly for women’s acceptance of that test to be highly correlated to institutional and provider support, suggesting that individual women are not making autonomous decisions about whether or not to accept the test [77]. Routinizing the offer and uptake of prenatal testing is problematic, since people’s desire for genomic information in the prenatal context has been shown to vary [78]. One study found that pregnant women who used an interactive decision-support guide were better informed of the benefits and risks of various prenatal testing modalities and were less likely to choose diagnostic testing, compared to women who received the standard of care [79]. Another study in which chromosome microarray was used to provide pregnant women with fetal genomic information found that, while women were initially enthusiastic about testing, many who received uncertain test results felt “blindsided,” described the results as “toxic knowledge,” and expressed regret at having undergone testing [80]. This was attributed at least in part to a lack of opportunity to weigh the benefits, risks, and consequences of testing [80]. The potential for routinization of NIPT is high, given its similarity to existing normalized prenatal tests and especially because of the simplicity of the procedure. If NIPT is routinized, women may not be provided with adequate opportunity to decline testing, or may not receive sufficient information to make an informed decision [76, 81], and may ultimately feel disempowered, rather than empowered, by their choice.

The worry around erosion of informed choice is a legitimate one: a previous study in the United Kingdom found that a small minority of clinicians believe the non-invasive, risk-free nature of NIPT means there is a less stringent requirement for informed choice [82]. Notwithstanding routinization, the current quality and quantity of information available raises concerns about informed decision making. We found that although women were satisfied with their understanding of NIPT [24, 25, 27], they demonstrated misunderstanding about certain aspects of the test, especially regarding the accuracy of NIPT [25, 27, 70], which conditions NIPT tests for [27], and the waiting period for return of results [27]. We also found that women were sometimes disappointed with the information their health care providers were able to provide [25, 34]. This is unsurprising given the range of sources from which clinicians learn about NIPT. A survey of 258 American obstetricians found that 36% of respondents first learned about NIPT from peer-reviewed publications, 36% from publications produced by professional organizations, and 28% from commercial laboratories [83]. More than that, 48% used commercial laboratories as their primary source of continued education about NIPT [83]. In some cases, the lack of standardized education on NIPT has led physicians to make errors when discussing it with patients, such as presenting NIPT as a diagnostic test rather than a screening test [84]. A recent study exploring the role of Dutch midwives as counsellors on NIPT found that only 59% were able to correctly answer seven or more questions out of eight standard knowledge questions about NIPT [85].

These discrepancies in clinician knowledge are an issue that needs to be remedied, however, there have been few suggestions for ameliorating this challenge. In a 2017 study, Oxenford and colleagues developed a training resource for health professionals offering NIPT in the United Kingdom, and evaluated the change in participants’ knowledge and confidence after a 40-min training session. [86]. Following the training session, there was a statistically significant increase in the number of participants who said they would feel confident discussing NIPT with patients, as well as a statistically significant increase in both perceived and actual knowledge about NIPT [86]. However, even after the training session, over 65% of participants still had some misconceptions, such as test turnaround time, false positive rates, whether the cell free fetal DNA originates from the placenta, and whether the concentration of fetal DNA in the bloodstream increases as the pregnancy progresses Martin and colleagues [85] also found significant knowledge discrepancies about NIPT among Dutch midwives, although noted that continuing education was positively correlated with an increase in participants’ knowledge about NIPT. It is clear that non-biased education resources in a variety of formats are needed to increase the confidence and comfort of health care providers counselling about NIPT.

In addition to increased education for providers, allocating more time to counselling may improve informed choice. Studies of women and clinicians identified strong consensus that consultations did not provide sufficient time for thorough counselling [30, 48, 87]. Some women found that their counselling session was too short for the volume of information they were presented with, and as a result felt overwhelmed [67]. A study among Dutch midwives has found that an appointment longer than 20 min – in some cases as long as 50 min – is sometimes required to provide adequate counselling [88]. However, the amount of time officially allocated for counselling may not correspond with the amount of time a clinician spends facilitating this discussion. One study in the Netherlands demonstrated that despite having 30 min of time allocated in the fee for counselling about prenatal testing, midwives spent on average 9 min conducting this discussion [85].

Women’s understanding has been shown to improve in a research setting, or a clinical setting with dedicated time and resources for counselling [89]. Outside of these settings, group prenatal counselling by expert clinicians has been suggested as a way to alleviate the barriers of time and provider confidence [90–92]. In the format proposed by Gammon and colleagues [90], several patients would receive education on prenatal screening and testing options at the same time, followed by a confidential, one-on-one session for individual questions and familial risk assessment. Gammon and colleagues [90] found that receiving counselling in this format increased participants’ acceptance of prenatal testing, their confidence in that decision, and their knowledge of the relevant technologies. This study provides us with an indication that group prenatal or genetic counselling sessions may be a viable option as we consider expanding access to NIPT. We are cautioned by the finding that group counselling increases uptake of prenatal testing, as this may indicate that the information provided or the social interaction of the group might make women feel pressured to test.

A small number of studies have indicated that the ease of access to NIPT, facilitated by the procedural simplicity may make women feel pressured to accept NIPT [93]. Public perception of NIPT as easy and risk-free was one source of pressure [24, 30, 93], but women also felt pressure from their family members and partners [30, 59]. Health care providers may also contribute to this pressure, as some approach NIPT with an attitude of “there is no downside” [94]. An additional worry associated with the procedurally simple, low-risk nature of NIPT, coupled with its high accuracy and early availability, is that it may be used for sex-selective termination [35, 36]. However, it is possible that ensuring women are well-informed about NIPT and other prenatal screening modalities may help to safeguard against such a use of the test. For example, Gammon and colleagues [90] found that participants who received group counselling were less likely to opt for screening for fetal sex.

A long standing concern about prenatal testing, exacerbated by the nature of NIPT is the potential for negative implications for the disabled community, as fewer people are born with conditions such as Down syndrome [39, 93, 95]. This is an enduring challenge that has been well described in the ethics literature [38, 77, 96, 97]. Many ethicists argue that the right of an individual woman to control her body and shape her family through informed autonomous choices takes priority over the more diffuse harm done to society when fewer people with particular conditions are born [98]. In this argument, the importance of informed autonomous choices is paramount. It is important that women have the opportunity to make decisions about reproduction based on their personal preferences, attitudes, and beliefs [72]. These decisions will differ – the availability of NIPT does not necessarily mean that more women will choose to terminate affected pregnancies. Some may decline all testing because they would never consider termination [68, 69]; others may decline NIPT and opt directly for invasive testing [27]; some choose to participate in NIPT in order to feel better prepared for the birth of an affected child [59]. Many mothers of children with Down syndrome would consider using NIPT if they become pregnant in the future, or would recommend NIPT to a pregnant friend [95]. Most (67%) also believe that NIPT should be available to all women [95]. When counselling women about NIPT, it is important that health care professionals provide patients with all of the information necessary to make an informed decision, in a non-biased manner, including the fact that declining prenatal testing is a valid option.

## Limitations

This synthesis of qualitative research builds a robust understanding of women’s preferences and experiences with making informed decisions about NIPT. While qualitative research findings are not intended to generalize directly to populations, the breadth of evidence synthesized here suggests insights which may be relevant to planning services in similar settings. Notably, all studies were conducted in high income jurisdictions, with highly-educated patient populations. Insights should be adapted accordingly when applied in other contexts.

## Conclusion

We examined 30 empirical primary qualitative research studies that describe women’s or their partner’s perspectives, experiences, and preferences about NIPT. From this body of evidence, we identified that women access a variety of sources to educate themselves about this technology, and that a women’s unique circumstances modulate the information that they value and require most in the context of making an informed decision. Although women are quite enthusiastic about NIPT, their discussions around ease of testing highlight threats to informed decision making such as routinization and or a pressure to test. Widened availability to trustworthy information about NIPT as well as careful attention to the facilitation of counselling may help safeguard informed decision-making.

## Additional file

Additional file 1: **Appendix 1.** Detailed literature search strategy. (DOCX 39 kb)

## Abbreviations

NIPT: Non-Invasive Prenatal Testing
SNP: Single-Nucleotide Polymorphism
