# Supplementary material for: Facilitating informed choice about non-invasive prenatal testing (NIPT): a systematic review and qualitative meta-synthesis of women’s experiences
Source: BMC Pregnancy Childbirth. 2019 Jan 14;19:27. doi: 10.1186/s12884-018-2168-4 (PMC6332899; doi:10.1186/s12884-018-2168-4)
Supplement: Supplementary file 1 — Appendix 1. Detailed literature search strategy. (DOCX 39 kb) [file 12884_2018_2168_MOESM1_ESM.docx]

**APPENDIX 1: LITERATURE SEARCH STRATEGIES**

Database: All Ovid MEDLINE(R) <1946 to Present>

Search Strategy:

--------------------------------------------------------------------------------

1     Sequence Analysis, DNA/ (145222)

2     ((DNA or parallel or next-generation or shotgun or target*) adj sequenc*).ti,ab,kf. (120098)

3     (MPSS or NGS or CSS or TMPS).ti,ab,kf. (13521)

4     High-Throughput Nucleotide Sequencing/ (18597)

5     ((high throughput adj2 (analys#s or sequenc*)) or single nucleotide polymorphism* or SNP or SNPs).ti,ab,kf. (111393)

6     or/1-5 (356702)

7     Genetic Testing/ (33131)

8     ((genetic* or gene*1 or genome*1 or genomic*) adj2 (test or tests or testing or diagnos#s or screen*)).ti,ab,kf. (56759)

9     or/7-8 (78355)

10     (noninvasive* or non-invasive*).ti,ab,kf. (164692)

11     9 and 10 (1050)

12     6 or 11 (357567)

13     Prenatal Diagnosis/ (35758)

14     ((antenatal or ante-natal or intrauterine or intra-uterine or prenatal or pre-natal) adj2 (test or tests or testing or diagnos#s or detect* or screen*)).ti,ab,kf. (34125)

15     (maternal adj2 (plasm* or blood)).ti,ab,kf. (12727)

16     or/13-15 (64934)

17     12 and 16 (2040)

18     (((f?etal or f?etus* or free-f?etal or placenta*) adj2 dna) or cell-free dna).ti,ab,kf. (3939)

19     (cff DNA or cffDNA or cf DNA or cfDNA or f DNA or fDNA or ff DNA or ffDNA).ti,ab,kf. (1337)

20     ((noninvasive* or non-invasive*) adj5 (prenatal or f?etal or f?etus*) adj (test or tests or testing or diagnos#s or detect* or screen*)).ti,ab,kf. (1568)

21     (NIPT or NIPD or NIDT or gNIPT or NIPS).ti,ab,kf. (1071)

22     or/17-21 (6854)

23     Qualitative Research/ (36358)

24     Interview/ (28094)

25     (theme$ or thematic).mp. (80191)

26     qualitative.af. (196605)

27     Nursing Methodology Research/ (16995)

28     questionnaire$.mp. (627589)

29     ethnological research.mp. (7)

30     ethnograph$.mp. (9033)

31     ethnonursing.af. (143)

32     phenomenol$.af. (22661)

33     (grounded adj (theor$ or study or studies or research or analys?s)).af. (9737)

34     (life stor$ or women* stor$).mp. (1169)

35     (emic or etic or hermeneutic$ or heuristic$ or semiotic$).af. or (data adj1 saturat$).tw. or participant observ$.tw. (19774)

36     (social construct$ or (postmodern$ or post-structural$) or (post structural$ or poststructural$) or post modern$ or post-modern$ or feminis$ or interpret$).mp. (475839)

37     (action research or cooperative inquir$ or co operative inquir$ or co-operative inquir$).mp. (3558)

38     (humanistic or existential or experiential or paradigm$).mp. (131602)

39     (field adj (study or studies or research)).tw. (14432)

40     human science.tw. (255)

41     biographical method.tw. (16)

42     theoretical sampl$.af. (566)

43     ((purpos$ adj4 sampl$) or (focus adj group$)).af. (50673)

44     (account or accounts or unstructured or openended or open ended or text$ or narrative$).mp. (551153)

45     (life world or life-world or conversation analys?s or personal experience$ or theoretical saturation).mp. (13987)

46     ((lived or life) adj experience$).mp. (8674)

47     cluster sampl$.mp. (5902)

48     observational method$.af. (633)

49     content analysis.af. (20465)

50     (constant adj (comparative or comparison)).af. (3786)

51     ((discourse$ or discurs$) adj3 analys?s).tw. (1823)

52     narrative analys?s.af. (947)

53     heidegger$.tw. (605)

54     colaizzi$.tw. (538)

55     spiegelberg$.tw. (81)

56     (van adj manen$).tw. (335)

57     (van adj kaam$).tw. (42)

58     (merleau adj ponty$).tw. (192)

59     husserl$.tw. (225)

60     foucault$.tw. (734)

61     (corbin$ adj2 strauss$).tw. (273)

62     glaser$.tw. (919)

63     or/23-62 (1985484)

64     22 and 63 (523)

65     limit 64 to (english language and yr="2007 -Current") (403)

EBSCOhost CINAHL

Top of Form

| **#** | **Query** | **Results** |
| --- | --- | --- |
| S1 | (MH "Sequence Analysis+") | 12,947 |
| S2 | ((DNA or parallel or next-generation or shotgun or target*) N1 sequenc*) | 3,719 |
| S3 | (MPSS or NGS or CSS or TMPS) | 1,680 |
| S4 | ((high throughput N2 (analys#s or sequenc*)) or single nucleotide polymorphism* or SNP or SNPs) | 8,019 |
| S5 | S1 OR S2 OR S3 OR S4 | 23,356 |
| S6 | (MH "Genetic Screening") | 9,422 |
| S7 | ((genetic* or gene or genes or genome* or genomic*) N2 (test or tests or testing or diagnos#s or screen*)) | 14,387 |
| S8 | S6 OR S7 | 14,387 |
| S9 | (MH "Noninvasive Procedures") | 1,781 |
| S10 | (noninvasive* or non-invasive*) | 22,544 |
| S11 | S9 OR S10 | 22,544 |
| S12 | S8 AND S11 | 295 |
| S13 | S5 OR S12 | 23,591 |
| S14 | (MH "Prenatal Diagnosis") | 6,278 |
| S15 | ((antenatal or ante-natal or intrauterine or intra-uterine or prenatal or pre-natal) N2 (test or tests or testing or diagnos#s or detect* or screen*)) | 9,741 |
| S16 | (maternal N2 (plasm* or blood)) | 1,823 |
| S17 | S14 OR S15 OR S16 | 11,235 |
| S18 | S13 AND S17 | 547 |
| S19 | (((f?etal or f?etus* or free-f?etal or placenta*) N2 dna) or cell-free dna) | 5,151 |
| S20 | (cff DNA or cffDNA or cf DNA or cfDNA or f DNA or fDNA or ff DNA or ffDNA) | 311 |
| S21 | ((noninvasive* or non-invasive*) N5 (prenatal or pre-natal or f?etal or f?etus*) N1 (test or tests or testing or diagnos#s or detect* or screen*)) | 1,085 |
| S22 | (NIPT or NIPD or NIDT or gNIPT or NIPS) | 410 |
| S23 | S18 OR S19 OR S20 OR S21 OR S22 | 6,499 |
| S24 | (MH "Interviews+") | 167,955 |
| S25 | MH audiorecording | 36,935 |
| S26 | MH Grounded theory | 12,160 |
| S27 | MH Qualitative Studies | 76,410 |
| S28 | MH Research, Nursing | 19,654 |
| S29 | MH Questionnaires+ | 305,524 |
| S30 | MH Focus Groups | 32,104 |
| S31 | MH Discourse Analysis | 3,572 |
| S32 | MH Content Analysis | 25,009 |
| S33 | MH Ethnographic Research | 6,166 |
| S34 | MH Ethnological Research | 5,200 |
| S35 | MH Ethnonursing Research | 180 |
| S36 | MH Constant Comparative Method | 6,388 |
| S37 | MH Qualitative Validity+ | 1,234 |
| S38 | MH Purposive Sample | 23,171 |
| S39 | MH Observational Methods+ | 18,407 |
| S40 | MH Field Studies | 2,539 |
| S41 | MH theoretical sample | 1,425 |
| S42 | MH Phenomenology | 2,624 |
| S43 | MH Phenomenological Research | 11,947 |
| S44 | MH Life Experiences+ | 25,258 |
| S45 | MH Cluster Sample+ | 3,495 |
| S46 | Ethnonursing | 258 |
| S47 | ethnograph* | 9,346 |
| S48 | phenomenol* | 17,535 |
| S49 | grounded N1 theor* | 14,278 |
| S50 | grounded N1 study | 1,539 |
| S51 | grounded N1 studies | 1,539 |
| S52 | grounded N1 research | 306 |
| S53 | grounded N1 analys?s | 467 |
| S54 | life stor* | 1,516 |
| S55 | women's stor* | 925 |
| S56 | emic or etic or hermeneutic* or heuristic* or semiotic* | 4,849 |
| S57 | data N1 saturat* | 434 |
| S58 | participant observ* | 9,712 |
| S59 | social construct* or postmodern* or post-structural* or post structural* or poststructural* or post modern* or post-modern* or feminis* or interpret* | 74,595 |
| S60 | action research or cooperative inquir* or co operative inquir* or co-operative inquir* | 7,282 |
| S61 | humanistic or existential or experiential or paradigm* | 27,631 |
| S62 | field N1 stud* | 4,551 |
| S63 | field N1 research | 1,446 |
| S64 | human science | 1,513 |
| S65 | biographical method | 56 |
| S66 | theoretical sampl* | 1,946 |
| S67 | purpos* N4 sampl* | 25,713 |
| S68 | focus N1 group* | 37,461 |
| S69 | account or accounts or unstructured or open-ended or open ended or text* or narrative* | 106,359 |
| S70 | life world or life-world or conversation analys?s or personal experience* or theoretical saturation | 8,542 |
| S71 | lived experience* | 5,213 |
| S72 | life experience* | 23,642 |
| S73 | cluster sampl* | 4,511 |
| S74 | theme* or thematic | 72,469 |
| S75 | observational method* | 18,576 |
| S76 | questionnaire* | 360,297 |
| S77 | content analysis | 31,142 |
| S78 | discourse* N3 analys?s | 4,124 |
| S79 | discurs* N3 analys?s | 180 |
| S80 | constant N1 comparative | 7,260 |
| S81 | constant N1 comparison | 964 |
| S82 | narrative analys?s | 2,113 |
| S83 | Heidegger* | 697 |
| S84 | Colaizzi* | 656 |
| S85 | Spiegelberg* | 26 |
| S86 | van N1 manen* | 534 |
| S87 | van N1 kaam* | 61 |
| S88 | merleau N1 ponty* | 156 |
| S89 | husserl* | 183 |
| S90 | Foucault* | 536 |
| S91 | Corbin* N2 strauss* | 267 |
| S92 | strauss* N2 corbin* | 267 |
| S93 | glaser* | 458 |
| S94 | S24 OR S25 OR S26 OR S27 OR S28 OR S29 OR S30 OR S31 OR S32 OR S33 OR S34 OR S35 OR S36 OR S37 OR S38 OR S39 OR S40 OR S41 OR S42 OR S43 OR S44 OR S45 OR S46 OR S47 OR S48 OR S49 OR S50 OR S51 OR S52 OR S53 OR S54 OR S55 OR S56 OR S57 OR S58 OR S59 OR S60 OR S61 OR S62 OR S63 OR S64 OR S65 OR S66 OR S67 OR S68 OR S69 OR S70 OR S71 OR S72 OR S73 OR S74 OR S75 OR S76 OR S77 OR S78 OR S79 OR S80 OR S81 OR S82 OR S83 OR S84 OR S85 OR S86 OR S87 OR S88 OR S89 OR S90 OR S91 OR S92 OR S93 | 754,462 |
| S95 | S23 AND S94 | 705 |
| S96 | Limiters - Published Date: 20070101-20171231   Narrow by Language: - english | 584 |

Bottom of Form

Social Sciences Citation Index

| Line # | Search | Notes |
| --- | --- | --- |
| 1 | TS=((DNA OR parallel OR next-generation OR shotgun OR target*) NEAR sequenc*) | Intervention |
| 2 | TS= (MPSS OR NGS OR CSS OR TMPS) |  |
| 3 | TS=((high throughput NEAR (analys?s OR sequenc*)) OR single nucleotide polymorphism* OR SNP OR SNPs) |  |
| 4 | #1 OR #2 OR #3 |  |
| 5 | TS=((genetic* OR gene OR genes OR genome* OR genomic*) NEAR (test OR tests OR testing OR diagnos?s OR screen*)) |  |
| 6 | TS=(noninvasive* or non-invasive*) |  |
| 7 | #5 AND #6 |  |
| 8 | #4 OR #8 |  |
| 9 | TS= ((antenatal OR ante-natal OR intrauterine OR intra-uterine OR prenatal OR pre-natal) NEAR (test OR tests OR testing OR diagnos?s OR detect* OR screen*)) |  |
| 10 | TS= (maternal NEAR (plasm* OR blood)) |  |
| 11 | #9 OR #10 |  |
| 12 | #8 AND #11 |  |
| 13 | TS=(((fetal OR faetal OR fetus* OR faetus* OR free-fetal OR free-faetal OR placenta*) NEAR dna) OR cell-free dna) |  |
| 14 | TS=(cff DNA OR cffDNA OR cf DNA OR cfDNA OR f DNA OR fDNA OR ff DNA OR ffDNA) |  |
| 15 | TS=((noninvasive* OR non-invasive*) NEAR (prenatal OR fetal OR faetal OR fetus OR faetus*) NEAR (test OR tests OR testing OR diagnos?s OR detect* OR screen*)) |  |
| 16 | TS=(NIPT OR NIPD OR NIDT OR gNIPT OR NIPS) |  |
| 17 | #12 OR #13 OR #14 OR #15 OR #16 | This line combines all the Intervention concepts, keywords |
| 18 | TS=interview* | Hybrid Filter: ISI Web of Science, Social Science Citation Index    Would recommend citing in methods/report:    DeJean D, Giacomini M, Simeonov D, Smith A. Finding Qualitative Research  Evidence for Health Technology Assessment. Qual Health Res. 2016  Aug;26(10):1307-17. |
| 19 | TS=(theme*) |  |
| 20 | TS=(thematic analysis) |  |
| 21 | TS=qualitative |  |
| 22 | TS=nursing research methodology |  |
| 23 | TS=questionnaire |  |
| 24 | TS=(ethnograph*) |  |
| 25 | TS= (ethnonursing) |  |
| 26 | TS=(ethnological research) |  |
| 27 | TS=(phenomenol*) |  |
| 28 | TS=(grounded theor*) OR TS=(grounded stud*) OR TS=(grounded research) OR TS=(grounded analys?s) |  |
| 29 | TS=(life stor*) OR TS=(women's stor*) |  |
| 30 | TS=(emic) OR TS=(etic) OR TS=(hermeneutic) OR TS=(heuristic) OR TS=(semiotic) OR TS=(data saturat*) OR TS=(participant observ*) |  |
| 31 | TS=(social construct*) OR TS=(postmodern*) OR TS=(post structural*) OR TS=(feminis*) OR TS=(interpret*) |  |
| 32 | TS=(action research) OR TS=(co-operative inquir*) |  |
| 33 | TS=(humanistic) OR TS=(existential) OR TS=(experiential) OR TS=(paradigm*) |  |
| 34 | TS=(field stud*) OR TS=(field research) |  |
| 35 | TS=(human science) |  |
| 36 | TS=(biographical method*) |  |
| 37 | TS=(theoretical sampl*) |  |
| 38 | TS=(purposive sampl*) |  |
| 39 | TS=(open-ended account*) OR TS=(unstructured account) OR TS=(narrative*) OR TS=(text*) |  |
| 40 | TS=(life world) OR TS=(conversation analys?s) OR TS=(theoretical saturation) |  |
| 41 | TS=(lived experience*) OR TS=(life experience*) |  |
| 42 | TS=(cluster sampl*) |  |
| 43 | TS=observational method* |  |
| 44 | TS=(content analysis) |  |
| 45 | TS=(constant comparative) |  |
| 46 | TS=(discourse analys?s) or TS =(discurs* analys?s) |  |
| 47 | TS=(narrative analys?s) |  |
| 48 | TS=(heidegger*) |  |
| 49 | TS=(colaizzi*) |  |
| 50 | TS=(spiegelberg*) |  |
| 51 | TS=(van manen*) |  |
| 52 | TS=(van kaam*) |  |
| 53 | TS=(merleau ponty*) |  |
| 54 | TS=(husserl*) |  |
| 55 | TS=(foucault*) |  |
| 56 | TS=(corbin*) |  |
| 57 | TS=(strauss*) |  |
| 58 | TS=(glaser*) |  |
| 59 | #18 OR #19 OR #20 OR #21 OR #22 OR #23 OR #24 OR #25 OR #26 OR #27 OR #28 OR #29 OR #30 OR #31 OR #32 OR #33 OR #34 OR #35 OR #36 OR #37 OR #38 OR #39 OR #40 OR #41 OR #42 OR #43 OR #44 OR #45 OR #46 OR #47 OR #48 OR #49 OR #50 OR #51 OR #52 OR #53 OR #54 OR #55 OR #56 OR #57 OR #58 | This line combines all the qualitative filter keywords |
| 60 | #17 AND #59 | Combine Intervention and filter |
